# Randomized Clinical Trial of Intravenous Valproate (Orifil) and Dexamethasone in Patients with Migraine Disorder

**Published:** 2013-06

**Authors:** Mohsen Foroughipour, Kavian Ghandehari, Mojtaba Khazaei, Fahimeh Ahmadi, Keyvan Shariatinezhad, Kosar Ghandehari

**Affiliations:** 1 Department of Neurology, Mashhad University of Medical Sciences, Mashhad, Iran;; 2 Cerebrovascular Research Center, Mashhad University of Medical Sciences, Mashhad, Iran;; 3 Department of Biostatistics, Mashhad University of Medical Sciences, Mashhad, Iran

**Keywords:** Valproic acid, Dexamethasone, Migraine disorders

## Abstract

**Background: **Intravenous Valproate (IVVP) has been used in the treatment of migraine in some studies; however, it is far better known in the management of status epilepticus.

**Methods: **Consecutive patients with migraine in our Headache Clinic were enrolled in this prospective, randomized clinical trial in 2011. The patients were randomized into two therapeutic groups, one receiving 900 mg IVVP (Orifil) and the other 16 mg IV Dexamethasone (IVDEX) diluted in 150 CC normal saline and infused for 10 minutes. Worst severity of pain before treatment and least severity at 3 hours after the infusion using a 0-10 point numeric rating scale were recorded. An interview with the patient was performed 72 hours after treatment to detect a possible relapse of headache.

**Results:** Thirty-one migraine status patients, comprising 28 women and 3 men at a mean±SD age of 33.355±12.373 SD, were investigated. Differences in the therapeutic effects of IVVP (Orifil) and IVDEX on pain score were not significant between the two groups (t=0.933, df=29; P=0.358). Relapse of headache occurred in 68.42% of the IVVP (Orifil) group and 66.67% of the IVDEX group. Distribution of relapse was not significantly different between the two therapeutic groups of patients (P=0.870).

**Conclusion: **IVVP (Orifil) was similar in efficacy to IVDEX as abortive therapy in patients with migraine status. IVVP (Orifil) appears to offer a safe and well-tolerated abortive treatment.

**Trial Registration Number:** IRCT13891146234N2

## Introduction

New Triptan drugs such as Almotriptan,^1^ and Lasmiditan,^2^ and other new drugs such as Telcageptant,^3^ and Tonabersat,^4^ have been developed for acute migraine management. Meanwhile, the treatment of migraine status remains a therapeutic challenge in some cases. There is an ongoing search for new treatment approaches for patients suffering from migraine and other headaches that are refractory to the usual abortive therapies.^5^

Oral Valproate is utilized widely as a preventive drug for migraine and for chronic daily headaches of migraine.^1^^,^^2^ Intravenous Valproate (IVVP) as abortive therapy has been administered in different doses for the management of migraine status in some studies.^6^^,^^7^ Many patients suffer prolonged, moderate, or severe migraines, and IVVP offers another weapon against these prolonged migraines. IVVP (Orifil) was studied for the treatment of migraine attacks in Israel,^8^ where the loading dose of IVVP (Orifil) was 900-1200 mg and the average time to best response for headache severity was 50 minutes. In addition, there was a significant reduction in pain severity (P<0.0001), and there were no serious adverse events. 

The ease of IVVP administration and absence of side effects are also important considerations; be that as it may, future double-blind studies will help clarify the situation.^9^ To review the literature regarding the use of intravenous valproic acid in aborting an acute migraine attack, a Medline (1967-June 2007) and bibliographic search of the English language literature was conducted using the search terms “valproic acid” and “migraine disorders”.^10^ All articles identified through the search were included.^10^ The use of intravenous valproic acid has been studied as a possible treatment for acute migraine.^10^ Available studies are small, mostly open-label and non-placebo-controlled, and used variable doses.^10^ Valproic acid has not been shown to be superior to comparator drugs.^10^ A few non-randomised and open lable studies were found, and intravenous valproate has shown better rsults in abortion of migraine attacks than placebo or comparating drugs.^10^ Future trials should be larger and placebo-controlled, and they should use a standardized dose and outcome measures. The present study is the first pilot study to compare the therapeutic effects between IVVP (Orifil) and IV Dexamethasone (IVDEX) in patients with migraine status.

## Methods

This prospective, controlled clinical trial recruited patients from our Emergency Division and Headache Clinic during 2011. Randomization was performed by a computerized software package. Neurologist and patient were blind to the selected therapeutic approach for each patient. Blinding was done by a research fellow. Diagnosis of migraine status was made by a neurologist according to the second edition of the International Headache Society (IHS) criteria,^11^ whereby migraine status was defined as a debilitating severe migraine attack lasting for more than 72 hours, and a present attack was that not attributable to another disorder. Interruption of headache during sleep and short lasting relief due to medication is disregarded disorder.^11^

Patients aged less than 18 years, pregnant women, and patients with liver failure were excluded.^12^ Patients with dementia, aphasia, and psychiatric disorders were also excluded. The severity of pain was classified based on the Pain Intensity Instrument, using a 0-to-10 point numeric rating scale.^13^ The patient was asked about what number on the 0-to-10 scale he/she would give for pain before treatment.^12^^,^^13^ Patients with migraine status were randomized into two therapeutic groups. An IV line was then established. In the first group, 16 mg IVDEX was diluted in 150 cc normal saline and infused for 10 minutes. (Patients at a minimum weight of 90 kg received 20 mg IVDEX.) The second group received 900 mg IVVP (Orifil) diluted in 150 cc normal saline and infused for 10 minutes. (Patients at a minimum weight of 90 kg received 1200 mg IVVP.) The patients were thereafter asked to rate the severity of their headache when it had the highest relief over a 3-hour period following the infusion.^12^ IVDEX has been the routine management of migraine status in our hospital in the recent decade. This standard of care in our hospital was fully explained to the patients; and if they agreed to receive IVVP, they were recruited in the case group**.** The worst severity of pain before treatment and the least severity over a 3-hour period after the infusion were recorded. The time to maximum relief and the time to onset of relief were recorded as well.^12^ Additionally, mean age, mean history of migraine, mean number of attacks per month, presence of aura, full recovery of headache post treatment, and recovery from nausea and photophobia post treatment were recorded in the questionnaire. Full recovery from headache post treatment was defined as pain-free response. The therapeutic effects of the drugs on the pain score, pre- and post-treatment periods, were defined as pain relief. An interview with the patient took place 72 hours after treatment to detect a possible relapse phenomenon.^13^ The relapse was categorized as mild and severe. Mild relapse was defined as recurrent headache requiring self-medication or no medication but not limiting activity, and severe relapse was defined as recurrent migraine attacks provoking another physician visit or interfering with daily activity.^14^

**Figure 1:**
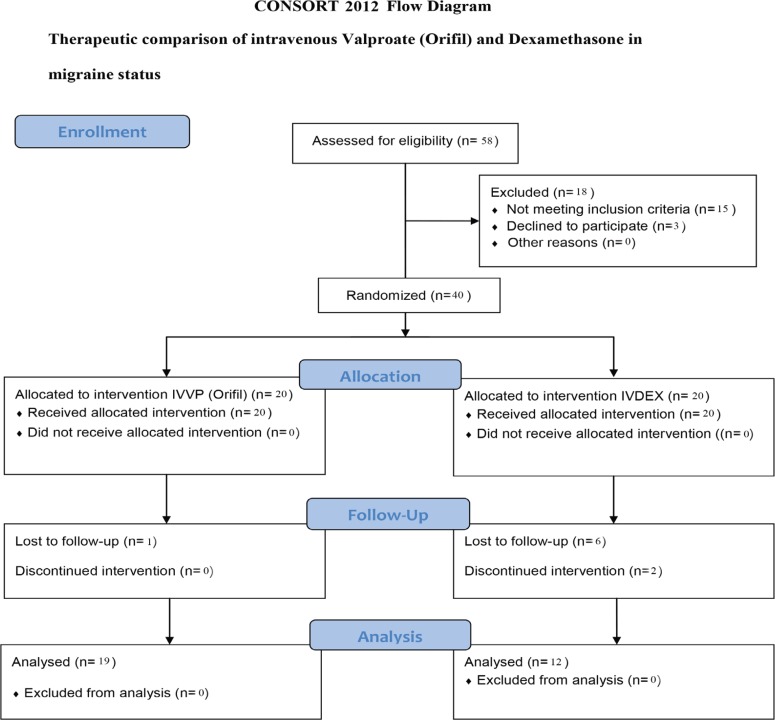
The patients’ consort flow chart is illustrated above

The research was approved by the local Ethics Committee (approval code number 1344), and an informed consent was obtained from all the patients. The patients’ CONSORT 2010 Flow Diagram is depicted in figure 1. Data on the patients’ demographics and above variables were recorded in a standardized questionnaire and entered in SPSS 16 software package. The parametric T test served for comparing mean age, mean history of migraine, mean duration of recovery onset, and mean duration of peak recovery effects between the two groups. Differences in the distribution of pain free response, recovery from photophobia and nausea, and recurrence patterns were analyzed using the Fisher exact test. 

## Results

Thirty-one migraine status patients, consisting of 28 women and 3 men with a mean age of 33.355, SD±12.373, were investigated. Nineteen cases (17 women, 2 men) received IVVP and 12 patients (11 women, one man) received IVDEX. All the patients had been taking preventive agents and abortive treatments. 

Table 1 illustrates the clinical characteristics of the patients and comparison thereof between the two therapeutic groups. The mean differences in pain score, pre- and post-treatment, periods between the IVVP and IVDEX groups were 5.789 (SD=3.44) and 6.833 (SD=2.209), respectively. The differences in the therapeutic effects of IVVP (Orifil) and IVDEX on pain score were not significant (t=0.933, df=29; P=0.358, mean difference=1.044, 95% CI: -1.244−3.331). The mean duration of recovery onset in the IVVP and IVDEX groups was 51.579 (SD=57.132) and 55.833 (SD=54.801) minutes, respectively; the differences in the mean duration of recovery onset between the two therapeutic groups were, however, not significant (t=0.205, df=29; P=0.839, mean difference=4.254, 95% CI: -38.175−46.684). The mean duration of peak recovery effect in the IVVP and IVDEX groups was 292.368 (SD=500.534) and 270.417 (SD=436.153) minutes, respectively, with the differences in the mean duration of peak recovery effect between the two therapeutic groups not constituting statistical significance (t=-0.125, df=29; P=0.902, mean difference=-21.952, 95% CI: -381.783−337.879). 

**Table 1:** Clinical characteristics of 31 migraine status patients and comparison thereof between the two therapeutic groups

**Clinical characteristic /Therapeutic group**	**IVVP Orifil** **N=19 **	**IV DEX ** **N=12**	**Statistical analysis**
	**mean** **±** **SD**	**mean** **±** **SD**	
Mean age*	33.895±13.337	32.500±11.188	t=-0.361, df=29, P=0.766
Mean migraine history **	6.263±8.993	6.000±8.068	t=-0.082, df=29, P=0.935
Presence of aura	38.84%	8.34%	P=0.077
Recovery from nausea	68.42%	91.67	P=0.201
Recovery from photophobia	78.95%	91.67%	P=0.624
Pain-free response after treatment	26.32%	33.33%	P=0.704

*Per year; **Per month; IVVP: intravenous Valproate; IVDEX: intravenous Dexamethasone; The t test was used for mean age and mean migraine history, and the Fisher test served for comparing the items in the four lower rows.

Table 2 illustrates the distribution of the recurrence patterns of migraine attacks in the two therapeutic groups within 72 hours after treatment. Relapse of headache occurred in 68.42% of the IVVP group and 66.67% of the IVDEX group within 72 hours; the distribution of the relapse patterns within 72 hours was not significantly different between the two therapeutic groups (P=0.870). None of the patients in the IVVP (Orifil) group or IVDEX group exhibited drug-related side effects within 72 hours post infusion.

**Table 2:** Distribution of the recurrence patterns of migraine attacks in the two therapeutic groups within 72 hours after treatment

**Recurrence pattern** **Number of patients**	**No recurrence**	**Mild recurrence**	**Severe recurrence**
IVVP(Orifil)	6	8	5
IVDEX	4	4	4
Total	10	12	9

IVVP: Intravenous Valproate; IVDEX: Intravenous Dexamethasone

## Discussion

The differences in terms of the pain relief effects of IVVP (Orifil) and IVDEX did not constitute statistical significance in our patients (P=0.358), denoting similarity in the therapeutic effects of IVVP and IVDEX in the treatment of migraine disorders. Other case series and open-label investigations, however, have documented clinically significant improvement of acute migraine headaches in patients treated with IVVP, particularly in a headache clinic setting.^14^^,^^15^ In one study, 85 patients with refractory migraine not responding to usual abortive treatments, including Triptans, Dihydroergotamine, and opioids, were treated with IVVP and the results demonstrated an 88% decrease in headache severity. In the study in question, the average dose of IVVP was 720 mg and the average time to best response was 50 minutes.^16^ In Czech Republic, 36 patients were prospectively treated in a non-randomized, open-label study to investigate the effectiveness of 500 mg IVVP in managing moderate to severe migraine headache. A meaningful reduction in headache within 2 hours was achieved in 20 out of 24 patients who had not been on oral Valproate prophylaxis and in all 12 patients in the subgroup with oral Valproate prophylaxis.^17^ In a US study, patients with severe migraine received a stat bolus of IVVP, immediately followed by an IV infusion of Methylprednisolone (500 mg) over a one-hour period, which was repeated every 3 weeks for one year. Among the 13 treated patients, 10 patients showed more than a 50% decline in the severity and frequency of pain.^15^ IVVP was also effective in the management of severe pediatric migraine in the US and 40% of those children experienced pain reduction.^18^ A clinical study in the US compared the therapeutic effects of Rizatriptan, Dexamethasone, and both in the acute treatment of menstrual migraine: in the assessment of 24-hour sustained pain relief and 24-hour sustained pain-free response, Rizatriptan was significantly superior to Dexamethasone and their combination was also superior to Rizatriptan and Dexamethasone separately.^19^ A Portuguese clinical study compared IVDEX (4 mg) and IV Haloperidol (5 mg) in the treatment of acute migraine: both drugs were equally efficient in pain relief after two hours.^20^ Another assessment of the effects of 300 mg IVVP in 61 Canadian patients with acute migraine revealed that 73% had significant pain relief in 30 minutes.^21^ However, in a placebo-controlled study of 126 patients with acute migraine headache, IVDEX (18 mg) failed to reduce headache relapses.^22^ A single 8 mg oral Dexamethasone following intravenous Phenothiazine treatment for migraine attack did not reduce the rate of recurrent headache in an Australian study.^23^ A placebo-controlled study was conducted by Fiesseler et al.^24^ on 173 patients with migraine. The authors did not find a statistically significant therapeutic effect of 10 mg IVDEX in patients with acute migraine headache.

The distribution of the relapse patterns was not significantly different between the two therapeutic groups in the present study (P=0.870). A pooled analysis of seven trials, involving 742 patients, suggested a modest but significant benefit in preventing headache recurrence when IVDEX was added to standard antimigraine therapy.^25^ Friedman et al.^26^ determined the efficacy of 10 mg IVDEX in a placebo-controlled study in 205 migrainous patients. Their study confirmed that a moderate dose of IVDEX should not be administered routinely in acute migraine, although it might be useful for patients with migraine status. Adding 3.5 mg of Prochlorperazine to 20 mg of IVDEX significantly shortened the response time for the abortive therapy of migraine status in one study.^27^ Elsewhere, the administration of IVDEX decreased the incidence of severe recurrent headache following the treatment of migraine attack,^14^ whereas it had no preventive effect on the recurrence of migraine in another study.^28^

The distribution of pain-free response was not significantly different between the IVVP and IVDEX groups of our patients (P**=**0.704). A meta-analysis of the effects of single-dose IVDEX, by comparison with a placebo, showed that IVDEX and placebo provided similar acute pain reduction; nevertheless, IVDEX was significantly more effective than the placebo in reducing headache recurrence within 72 hours.^29^ Another US study included 40 participants with prolonged severe migraine, who alternatively received either 500 mg IVVP or 10 mg Metoclopramide intramuscularly, followed by one mg Dihydroergotamine. The study reported that IVVP was similar in effectiveness to Metoclopramide/Dihydroergotamine as abortive therapy for prolonged severe migraine.^30^ It is deserving of note that although textbooks on headache have mentioned the usefulness of IVVP in the treatment of severe acute migraine,^31^ further research is required to shed sufficient light on this issue.

## Conclusion

A meticulous review of the literature shows that our study is the only clinical trial to date to compare 16 mg IVDEX with 900 mg IVVP (Orifil) in patients with migraine status. In our study, IVVP (Orifil) was similar to IVDEX as abortive therapy and IVVP (Orifil) appears to offer a safe and well-tolerated abortive treatment. 
